# Comparison of advanced techniques for local excision of rectal lesions: a case series

**DOI:** 10.1186/s12893-022-01543-w

**Published:** 2022-03-27

**Authors:** Marisa E. Schwab, Sophia Hernandez, Sarah Watanaskul, Hueylan Chern, Madhulika Varma, Ankit Sarin

**Affiliations:** 1 Department of Surgery, University of California San Francisco, San Francisco, CA USA; 2 School of Medicine, University of California San Francisco, San Francisco, CA USA

**Keywords:** Rectal lesion, Local excision, Robotic surgery, Transanal minimally invasive surgery

## Abstract

**Background:**

Robotic transanal minimally invasive surgery (R-TAMIS) is an appealing alternative to transanal minimally invasive surgery (TAMIS) and transanal endoscopic microsurgery (TEM) for benign and early malignant rectal lesions that are not amenable to traditional open transanal excision. However, no studies to our knowledge have directly compared the three techniques. This study sought to compare peri-operative and pathologic outcomes of the three approaches.

**Methods:**

The records of 29 consecutive patients who underwent TEM, TAMIS, or R-TAMIS at a single academic center between 2016 and 2020 were reviewed. Intra-operative details, pathological diagnosis and margins, and post-operative outcomes were recorded. The three groups were compared using chi-square and Kruskal–Wallis tests.

**Results:**

Overall, 16/29 patients were women and the median age was 57 (interquartile range (IQR): 28–81). Thirteen patients underwent TEM, six had TAMIS, and 10 had R-TAMIS. BMI was lower in the R-TAMIS patients (24.7; IQR 23.8–28.7), than in TEM (29.3; IQR 19.9–30.2), and TAMIS (30.4; IQR 26.6–32.9) patients. High grade dysplasia and/or invasive cancer was more common in TAMIS (80%) and R-TAMIS (66.7%) patients than in TEM patients (41.7%). The three groups did not differ significantly in tumor type or distance from the anal verge. No R-TAMIS patients had a positive surgical margin compared to 23.1% in the TEM group and 16.7% in the TAMIS group. Length of stay (median 1 day for TEM and R-TAMIS patients, 0 days for TAMIS patients) and 30-day readmission rates (7.7% of TEM, 0% of TAMIS, 10% of R-TAMIS patients) also did not differ among the groups. Median operative time was 110 min for TEM, 105 min for TAMIS, and 76 min for R-TAMIS patients.

**Conclusions:**

R-TAMIS may have several advantages over other advanced techniques for transanal excisions. R-TAMIS tended to be faster and to more often result in negative surgical margins compared to the two other techniques.

**Supplementary Information:**

The online version contains supplementary material available at 10.1186/s12893-022-01543-w.

## Background

Transanal local excision is indicated for benign rectal lesions, malignant rectal T1 adenocarcinomas that are moderately and well differentiated, without lymphovascular or perineural invasion, and small neuroendocrine tumors of the rectum [1]. When traditional open transanal excision is not feasible because the lesion is located in the mid to proximal rectum, two other techniques are transanal endoscopic microsurgery (TEM) and laparoscopic transanal minimally invasive surgery (TAMIS). More recently, robotic transanal minimally invasive surgery (R-TAMIS) has become an increasingly appealing alternative. Although the benefits of transabdominal pelvic robotic surgery over laparoscopic surgery are just emerging [2, 3] whether these benefits translate to transanal robotic surgery is unclear. Another advantage of robotic surgery is improved surgeon ergonomics during pelvic and abdominal surgeries [4, 5], but whether this holds for transanal robotic surgery is not well established.

Robotic surgery for transanal excision of rectal lesions is a relatively new approach. In the largest series to date at a single institution, 58 patients underwent R-TAMIS, 55 of whom had negative surgical margins. Of those 55, three patients developed local recurrence at a mean follow-up of 12 months, requiring salvage surgery [6]. The authors concluded that R-TAMIS improved surgeon ergonomics and enabled tumor removal from all quadrants [6]. There is still a lack of studies directly comparing R-TAMIS, TAMIS, and TEM [7]. We sought to address this gap by comparing operative, perioperative, and pathological outcomes of these three distinct approaches at a single tertiary academic institution.

## Methods

Data from all adult patients (> 21 years old) who underwent advanced transanal excision of a rectal lesion at a single academic center (University of California San Francisco) were retrospectively collected from a database of consecutive patients undergoing colorectal surgery from 2016 to 2020. In our clinical practice, patients were considered for advanced transanal excision if lesions were not amenable to traditional transanal excision and were one of the following: (1) the biopsy of the lesion was benign (adenomatous polyps) with no evidence of deep invasion on MRI, or (2) the biopsy showed rectal adenocarcinoma which, on preoperative magnetic resonance imaging (MRI) or endoscopic ultrasound (EUS) was read as a T1/T2 N0 lesion, or (3) rectal neuroendocrine tumors < 2 cm. [1] For those in whom pre-operative MRI was unable to distinguish between T1 and T2, patients underwent a local excision with the understanding that a T2 lesion on final pathology would require a subsequent radical resection. All lesions had to be within 13 cm of the anal verge. Two colorectal surgeons (CH, AS) performed all the operations. Before May 2018, all patients were offered TEM. From May 2018 to December 2019, patients were offered TAMIS, and from January 2020, all patients were offered R-TAMIS with the Da Vinci Robotic Surgical System Xi (Intuitive Surgical, Inc., Sunnyvale, CA). Thus, no selection criteria were used to determine use of one technique versus another; the surgical approach received was dependent on when the patient presented to our practice. The R-TAMIS procedure started at proctoscopy, followed by the Gelpoint being placed and the robot being docked.

Patients’ electronic medical records were retrospectively reviewed for demographics and clinical diagnosis; intra-operative details such as operative time, lesion location and size; pathological diagnosis, intactness of specimen, and margins; and post-operative complications and outcomes with follow-up time ranging from 3 months to 5 years. The direct cost of each operation was collected from the hospital’s cost accounting management system, linked to the patient’s encounter within the electronic medical record system.

Data are summarized as medians and interquartile ranges (IQR). The three groups were compared using the chi-square test for categorical variables and the Kruskal–Wallis test for continuous variables. Statistical significance was defined as a p ≤ 0.05. Statistical analyses were completed using Stata/IC version 16.1 (StataCorp, LLC, College Station, TX).

A video compilation was created of some of the robotic cases included in this study, to illustrate the key steps of this operation in different types of tumors that can be resected by R-TAMIS. This study was approved by the Institutional Review Board at the University of California, San Francisco (UCSF): Study Number 18-26677.

## Results

### Patient demographics

A total of 29 patients were included in our review: 13 TEM, 6 TAMIS, 10 R-TAMIS (Table 1). A video compilation of the key steps in four different patients treated with R-TAMIS is presented in Additional file 1. The median cohort age was 57 (IQR 28–81) and there were 16 women. Twenty-two (76%) patients identified as White. There were no significant demographic differences among the three groups. Patients who underwent R-TAMIS tended to have a lower body mass index (BMI) than patients in the other groups. American Society of Anesthesiologist (ASA) classification differed significantly: most TAMIS patients were ASA class 3, whereas most TEM and R-TAMIS patients were ASA Class 2 (p = 0.004). One hundred percent of the TAMIS and R-TAMIS patients underwent pre-operative antibiotic bowel preparation, whereas only 61.5% of TEM patients did so (p = 0.02).

**Table 1:** Demographics of 29 patients who underwent TEM, TAMIS, or R-TAMIS

	TEM (n = 13)	TAMIS (n = 6)	R-TAMIS (n = 10)	
Variables	Median (IQR) or Frequency (%)	Median (IQR) or Frequency (%)	Median (IQR) or Frequency (%)	*P*-value
Age	56 (51–70)	56 (52–62)	58 (50–65)	0.28
Sex, male	6 (46.2)	2 (33.3)	5 (50.0)	0.80
Race				0.83
White	10 (76.9)	4 (66.7)	8 (80.0)	
Non-white	3 (23.1)	2 (33.3)	2 (20.0)	
Average BMI (kg/m^2^)	29.3 (19.9–30.2)	30.4 (26.6–32.9)	24.7 (23.8–28.7)	0.29
ASA class				0.004*
1	0 (0.0)	0 (0.0)	2 (20.0)	
2	11 (84.6)	1 (16.7)	7 (70.0)	
3	2 (15.4)	5 (83.3)	1 (10.0)	
Mechanical bowel prep	6 (50.0)	4 (80.0)	7 (77.8)	0.31
Antibiotic bowel prep	8 (61.5)	6 (100.0)	10 (100.0)	0.02*

*BMI* body mass index, *ASA* American Society of Anesthesiologists classification, *IQR* interquartile range, * indicates statistical significance

### Tumor characteristics

A higher proportion of patients who underwent TAMIS (80%) and R-TAMIS (66.7%) had high grade dysplasia or invasive cancer than those who had TEM (41.7%), though the difference was not statistically significant. Tumor type or distance from the anal verge did not differ significantly among the three procedures (Table 2). Positive surgical margins were seen in 3/13 of the TEM patients, 1/6 of TAMIS and 0/10 of the R-TAMIS patients (Table 3).

**Table 2:** Tumor characteristics among the three groups

	TEM (n = 13)	TAMIS (n = 6)	R-TAMIS (n = 10)	
Variables	Median (IQR) or Frequency (%)	Median (IQR) or Frequency (%)	Median (IQR) or Frequency (%)	*P*-value
Degree of dysplasia				0.29
Benign/LGD	7 (58.3)	1 (20.0)	2 (33.3)	
HGD/Invasive	5 (41.7)	4 (80.0)	4 (66.7)	
Tumor pathology				0.21
Adenocarcinoma	4 (40.0)	1 (20.0)	3 (42.9)	
Tubular Adenoma	4 (40.0)	1 (20.0)	4 (42.9)	
HSIL/SIL	0 (0.0)	2 (40.0)	0 (0.0)	
Neuroendocrine	1 (10.0)	1 (20.0)	0 (0.0)	
Serrated adenoma	1 (10.0)	0 (0.0)	0 (0.0)	
Villous adenoma	0 (0.0)	0 (0.0)	1 (10.0)	
Benign polyp/nodule	1 (10.0)	0 (0.0)	0 (0.0)	
Tumor location				0.09
Anterior	4 (30.8)	0 (0.0)	1 (10.0)	
Lateral	4 (30.8)	3 (75.0)	3 (30.0)	
Posterior	4 (30.8)	0 (0.0)	6 (60.0)	
Anterior/Posterior + Lateral	1 (7.7)	1 (25.0)	0 (0.0)	
Distance from anal verge (cm)	5 (4–8)	6 (6–8)	8 (7–10)	0.27
Positive margins	3 (23.1)	1 (16.7)	0 (0.0)	0.28

*LGD* low-grade dysplasia, *HGD* high-grade dysplasia, *HSIL* high-grade squamous intraepithelial lesion, *SIL* squamous intraepithelial lesion, IQR, interquartile range

**Table 3:** Intra-operative details and post-operative outcomes among the three groups

	TEM (n = 13)	TAMIS (n = 6)	R-TAMIS (n = 10)	
Variables	Median (IQR) or Frequency (%)	Median (IQR) or Frequency (%)	Median (IQR) or Frequency (%)	*P*-value
Time in OR (min)	245 (213–317)	273 (262–280)	241 (208–286)	0.83
Procedure duration (min)	110 (78–136)	105 (96–112)	76 (51–101)	0.20
Positioning				0.49
Lithotomy	5 (38.5)	3 (60.0)	6 (60.0)	
Prone	4 (30.8)	2 (40.0)	4 (40.0)	
Supine	1 (7.7)	0 (0.0)	0 (0.0)	
Lateral decubitus	3 (23.8)	0 (0.0)	0 (0.0)	
EBL (mL)	10 (10–20)	15 (5–50)	5 (5–50)	0.63
Direct Cost (USD)	6362 (5286–6721)	6428 (5177–8060)	9226 (7835–10,224)	0.60
Total OME during Hospitalization	0 (0–3.8)	0 (0–0)	0 (0–15)	0.81
Length of stay (days)	1 (1–1)	0.5 (0–1)	1 (0–1)	0.60
30 day readmission	1 (7.7)	0 (0.0)	1 (10.0)	0.74

*EBL* estimated blood loss, *OME* oral morphine equivalents, *IQR* interquartile range

**Fig. 1:**
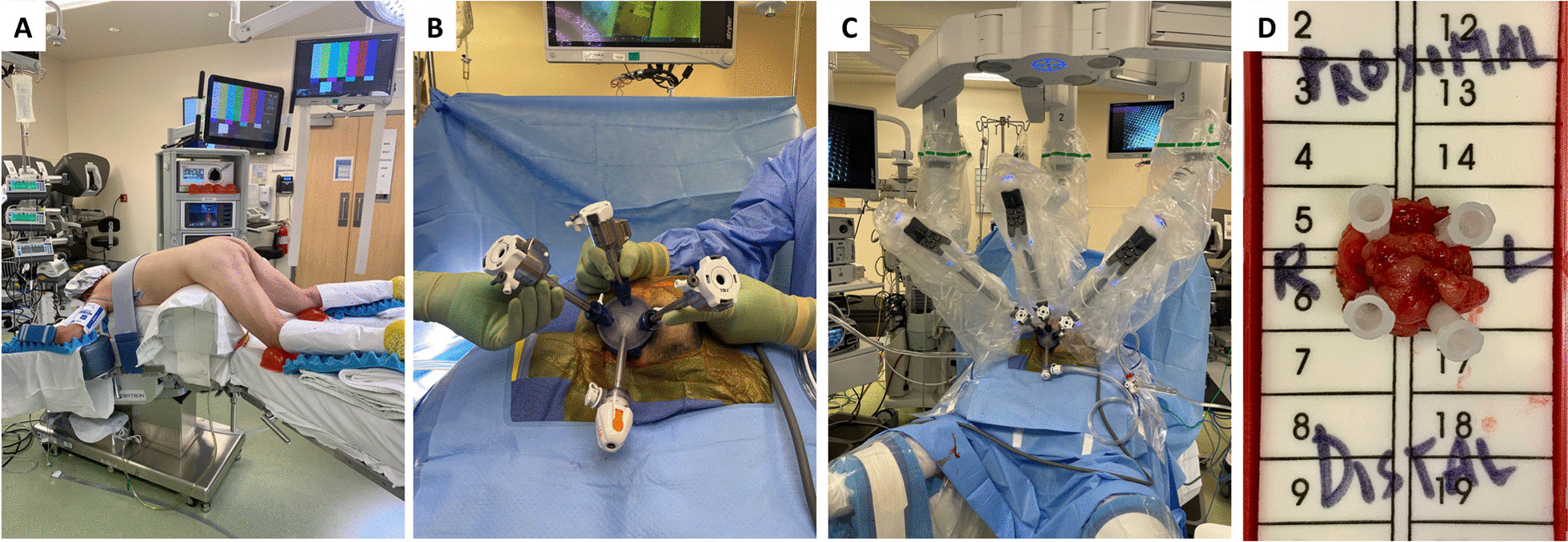
Intra-operative photos of transanal rectal excision using R-TAMIS. **A** Patient positioned prone jackknife. **B** Set-up of Gelpoint path port, through which the robotic ports are placed, and monitor. **C** Robotic trocars and airseal insufflator placement through Gelport. **D** Rectal lesion specimen oriented for pathological evaluation

*Intra-operative details*: The median time in the operating room and the median procedure duration was lowest in the R-TAMIS group (Table 3). Forty percent of the TAMIS and R-TAMIS patients were placed in prone position versus 30.8% of the TEM patients (Fig. 1). The direct cost was higher in the R-TAMIS group ($9226, IQR $7835–$10,224) than in the other groups ($6362, IQR $5286–$6721 in TEM, $6428, IQR $5177–$8060 in TAMIS).

### Post-operative outcomes

Postoperative length of stay did not differ among the groups (Table 3). One patient in the TEM group was readmitted for pain control. One patient in the TAMIS group was readmitted due to a concern for a pelvic abscess, was brought back to the OR, and no abscess was found. In the TEM group, 4/13 patients required further surgery: one patient was found to have a T2 lesion on final pathology and underwent a robotic low anterior resection (LAR), one patient had positive surgical margins and was recommended to follow up to discuss surgical options but was lost to follow up, one patient’s TEM was aborted due to the location and underwent an LAR one month later, and one patient had a recurrence one year later thus was scheduled for an abdominoperineal resection (APR). In the TAMIS group, 4/6 patients required further surgery: two patients were found to have a T2 lesion on final pathology and underwent LAR, one patient developed a rectal stricture requiring exams under anesthesia with dilations, and one patient with HSIL in the setting of ulcerative colitis underwent a total colectomy one year later. In the R-TAMIS group, 1/10 patients was found to have a T2 lesion on final pathology and was scheduled for an LAR.

## Discussion

As robotic TAMIS gains traction as a technique for transanal excision of benign and early malignant rectal lesions, a handful of series have described the outcomes [6–10], but none compared R-TAMIS to TAMIS and TEM. Our study directly compared these three groups at a single tertiary academic institution with respect to perioperative and pathological outcomes. The results suggest a tendency for R-TAMIS to be faster, have a higher frequency of negative surgical margins, and lower requirement for further surgery, compared to TEM and laparoscopic TAMIS. We present a video compilation of different R-TAMIS cases to illustrate the benefits of this technique for different types of tumor.

In terms of patient positioning, our experience with R-TAMIS is similar to that of other authors. Case series of patients undergoing R-TAMIS for rectal lesions have described several advantages including better 3D visualization, improved surgeon ergonomics, and the ability to position the patient in a consistent position regardless of the tumor location [11]. This is due to the Da Vinci’s 7 degrees of freedom, which enable the circumferential resection of lesions regardless of patient positioning. Authors of a study of two tertiary referral centers advocated for the lithotomy position (used in 32/34 of their patients) because of the additional time needed to position the patient in a prone or jackknife position [9]. In our study, 40% of patients in all three groups were in the prone position. We found a non-significant tendency for lower operative time in the R-TAMIS group, which leads us to recommend considering jackknife positioning for R-TAMIS. The advantage of using the prone approach is that the legs of the patient do not interfere with the positioning of the robotic arms and there is less risk of positioning or mechanical injury to the legs. Although the robot enables the surgeon to operate in all quadrants, we believe that the operation is faster and more straightforward if the lesion is positioned to be inferior on the screen. Others have expressed theoretical concerns for anesthetic concerns in jackknife positioning especially if the patient is under general anesthesia [7], but this has not been our experience. Moreover, the safety of this positioning for anorectal surgeries of similar duration has been well established. [12].

With R-TAMIS, we could reach tumors at a median 8 cm from the anal verge. Compared to TEM, which is rigid and therefore allows only a limited field of view at one time making excision of large lesions difficult and closure of large defects challenging, the robotic platform allows 360 degrees of view due to its flexible platform. In addition, the robot aids in suturing high in the rectum as it does not ergonomically limit the surgeon at the console even though instrument movement may be somewhat restricted similar to TEM or L-TAMIS. Others have also advocated for the ability to reach very low-lying tumors due to the articulation of the wrist [11] although these lesions may not require any advanced instrumentation. None of our robot excisions required conversion to a different approach, a finding similar to the experience of others [9]. Our median operative time of 76 min and median distance of the tumor from the anal verge (8 cm) were also similar to what others have reported. [6]

In our study, pre-operative antibiotic bowel preparation was significantly more common in the TAMIS and R-TAMIS groups than in the TEM group. This is likely a reflection of how practices have evolved over time. It is encouraging that patients in all three groups received few to no opioids during their hospitalization.

We also found no positive surgical margins in the R-TAMIS group. In other case series of only R-TAMIS patients, negative margin rates have ranged from 0% [11] to 8.7% [8]. In our series, while limited by the small number of patients, there was no significant difference in positive margins between the three groups. In other larger studies of each technique, TEM was associated with up to 17% rate of positive margins [13] and TAMIS with 7% [14]. However, only our study directly compared the three techniques.

The median direct cost of R-TAMIS in our study was higher than that of TEM and TAMIS, although this was not statistically significant (median $9,226 in R-TAMIS group, $6,362 in TEM group, and $6,428 in TAMIS group). Another study that compared the cost of R-TAMIS to TAMIS also found that the median direct cost was unsurprisingly higher in R-TAMIS ($4441 versus $3562) [15]. The median length of stay in all three of our groups was one day, but the significantly lower number of patients requiring further surgery after R-TAMIS due to better margins may offset the higher direct cost of the initial operation. We found no significant differences in 30 day readmissions among the three groups.

The limitations of this study include the small sample size and retrospective design. The primary surgeon had completed 30 prior transanal procedure at another institution prior to this work and the techniques were introduced based on available technology. As such the role of learning curve was minimal. There was no selection bias towards robotic surgery in this study as any patients who met criteria for a transanal excision was offered surgery and the technique offered was based on the available technology at the time. No selection towards one or other technique was made based on patient characteristics. The short follow-up time also limits our knowledge of the long-term oncologic and functional outcomes. The BMI of the R-TAMIS group tended to be lower than in the other groups, which may have contributed to the shorter operative time for that group. Others have found that a higher BMI was associated with longer R-TAMIS times. [9]

## Conclusions

Our comparison of R-TAMIS, TAMIS, and TEM at a single tertiary academic institution shows that R-TAMIS may have several advantages over conventional endoscopic and laparoscopic excision of transanal lesions. Larger studies with longer follow-up times are needed to confirm these findings and clarify the role of robotic TAMIS in the care of patients with rectal lesions.

## Supplementary Information

**Additional file 1. **R-TAMIS video.

## Data Availability

The datasets used and/or analysed during the current study are available from the corresponding author on reasonable request.

## Abbreviations

ASA: American Society of Anesthesiologist classification
BMI: Body mass index
OME: Oral morphine equivalents
IQR: Interquartile range
R-TAMIS: Robotic transanal minimally invasive surgery
TAMIS: Transanal minimally invasive surgery
TEM: Transanal endoscopic microsurgery
